# Restoration experiments in polymetallic nodule areas

**DOI:** 10.1002/ieam.4541

**Published:** 2021-11-10

**Authors:** Sabine Gollner, Matthias Haeckel, Felix Janssen, Nene Lefaible, Massimiliano Molari, Stavroula Papadopoulou, Gert‐Jan Reichart, João Trabucho Alexandre, Annemiek Vink, Ann Vanreusel

**Affiliations:** ^1^ Department of Ocean Systems Royal Netherlands Institute for Sea Research (NIOZ) Den Burg the Netherlands; ^2^ GEOMAR Helmholtz Center for Ocean Research Kiel Kiel Germany; ^3^ HGF MPG Joint Research Group for Deep Sea Ecology and Technology Alfred Wegener Institute Helmholtz Centre for Polar and Marine Research (AWI) Bremerhaven Germany; ^4^ HGF MPG Joint Research Group for Deep Sea Ecology and Technology Max Planck Institute for Marine Microbiology (MPI) Bremen Germany; ^5^ Marine Biology Research Group Ghent University Ghent Belgium; ^6^ WerkStaat Heidelberg Germany; ^7^ Department of Earth Sciences Utrecht University Utrecht the Netherlands; ^8^ Federal Institute for Geosciences and Natural Resources (BGR) Hannover Germany

**Keywords:** Artificial nodules, Deep sea, Minerals, Mining, Mitigation

## Abstract

Deep‐seabed polymetallic nodule mining can have multiple adverse effects on benthic communities, such as permanent loss of habitat by removal of nodules and habitat modification of sediments. One tool to manage biodiversity risks is the mitigation hierarchy, including avoidance, minimization of impacts, rehabilitation and/or restoration, and offset. We initiated long‐term restoration experiments at sites in polymetallic nodule exploration contract areas in the Clarion‐Clipperton Zone that were (i) cleared of nodules by a preprototype mining vehicle, (ii) disturbed by dredge or sledge, (iii) undisturbed, and (iv) naturally devoid of nodules. To accommodate for habitat loss, we deployed >2000 artificial ceramic nodules to study the possible effect of substrate provision on the recovery of biota and its impact on sediment biogeochemistry. Seventy‐five nodules were recovered after eight weeks and had not been colonized by any sessile epifauna. All other nodules will remain on the seafloor for several years before recovery. Furthermore, to account for habitat modification of the top sediment layer, sediment in an epibenthic sledge track was loosened by a metal rake to test the feasibility of sediment decompaction to facilitate soft‐sediment recovery. Analyses of granulometry and nutrients one month after sediment decompaction revealed that sand fractions are proportionally lower within the decompacted samples, whereas total organic carbon values are higher. Considering the slow natural recovery rates of deep‐sea communities, these experiments represent the beginning of a ~30‐year study during which we expect to gain insights into the nature and timing of the development of hard‐substrate communities and the influence of nodules on the recovery of disturbed sediment communities. Results will help us understand adverse long‐term effects of nodule removal, providing an evidence base for setting criteria for the definition of “serious harm” to the environment. Furthermore, accompanying research is needed to define a robust ecosystem baseline in order to effectively identify restoration success. *Integr Environ Assess Manag* 2022;18:682–696. © 2021 The Authors. *Integrated Environmental Assessment and Management* published by Wiley Periodicals LLC on behalf of Society of Environmental Toxicology & Chemistry (SETAC).

## Key Points

- Adverse effects on benthic communities as expected from deep‐seabed polymetallic nodule mining call for the application of the mitigation hierarchy—including restoration measures once feasibility and positive effects have been proven.
- We initiated long‐term restoration experiments at sites in polymetallic nodule exploration contract areas in the Clarion‐Clipperton Zone that were (i) cleared of nodules by a preprototype mining vehicle, (ii) disturbed by dredge or sledge, (iii) undisturbed, and (iv) naturally devoid of nodules.
- We deployed >2000 artificial ceramic nodules to study the possible effect of substrate provision on recovery of biota and its impact on sediment biogeochemistry, and loosened sediment with a metal rake to test the feasibility of sediment decompaction to facilitate soft‐sediment recovery.
- Due to expected slow recovery rates and the need for detailed characterization of baseline conditions, we expect to gain conclusive knowledge on long‐term adverse effects of nodule removal and on the effectiveness of restoration measures in the next 30 years.

## INTRODUCTION

Deep‐sea polymetallic nodules are seen as an upcoming potential resource for critical metals to support growing populations, urbanization, high technology applications, and the development of a green energy economy (Hein et al., 2020). At the same time, mineral exploitation may threaten the future integrity of deep‐sea ecosystems (Drazen et al., 2020; Levin et al., 2020; Niner et al., 2018; Smith et al., 2020). Regulators are currently establishing governance frameworks for sustainable management of minerals and other deep‐sea resources, while scientists are rapidly making new discoveries on biodiversity, ecosystem functions, and resilience of deep‐sea communities. Yet, there is very little knowledge on the potential of ecological restoration in the deep sea to mitigate impacts, despite the fact that the communities of large parts of the seafloor may undergo significant disturbance in the (near) future.

**Figure 1:**
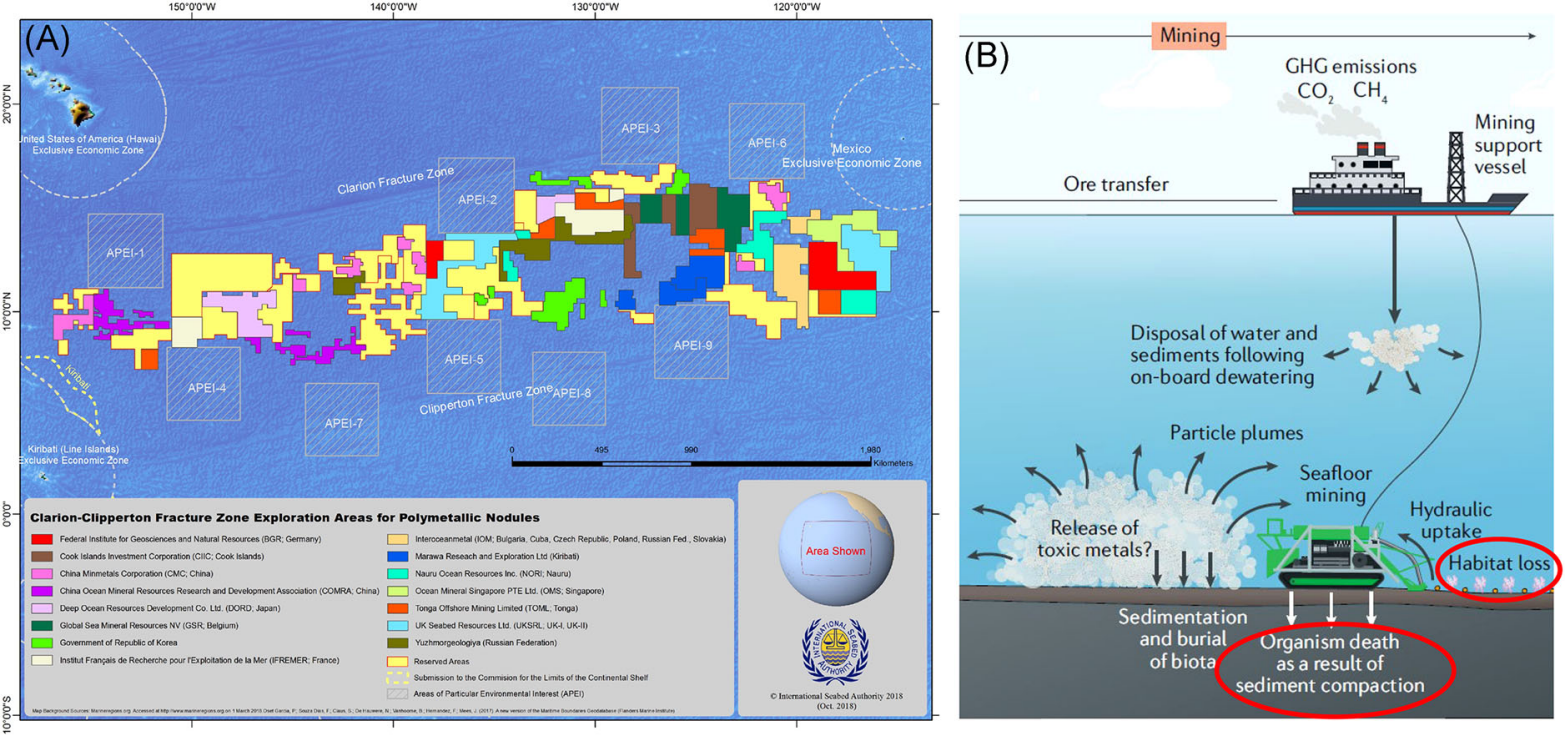
(A) Map showing the contract areas for polymetallic nodule exploration and Areas of Particular Environmental Interest (APEI) in the Clarion‐Clipperton Zone (CCZ) (© ISA). (B) Expected impacts of deep‐seabed mining. Red circles indicate impacts addressed in our scientific restoration experiments. Figure slightly modified after Hein et al. (2020) (excluding transportation and exploration impact). Note that the return water (dewatering plant) sediment plume may be close to the seafloor instead of midwater. Parts of 1B reprinted by permission from Copyright Clearance Center: Springer Nature, Nature Reviews Earth & Environment, Deep‐ocean polymetallic nodules as a resource for critical materials (Hein et al., 2020)

For the seabed beyond national jurisdiction, 19 exploration contracts for polymetallic nodules have been awarded by the International Seabed Authority (ISA), each area covering 75 000 km^2^, totaling up to 1 425 000 km^2^ of the seabed (www.isa.org.jm). The region of main interest is the Clarion‐Clipperton Zone (CCZ), situated in the Pacific Ocean (Figure 1A). Potential physical impacts of nodule mining are numerous, and include, for example, removal of substrates (nodules and sediment), alteration of habitat, and the spread and redeposition of sediment plumes created by the mining vehicle (Boetius & Haeckel, 2018; Gollner et al., 2017; Miller et al., 2018) (Figure 1B). The most long‐lasting physical impact may be the removal of nodules from the sediment surface, as nodules form at rates of only a few millimeters per million years (Lyle, 1982). Recovery of the physical nodule environment at a mined site, therefore, takes millions of years. In addition, changes to surface sediments (compaction, sediment removal, and sediment redeposition with different physical and biogeochemical properties) will also be long‐term, as natural sedimentation rates in the CCZ are less than 1 cm per thousand years (Volz et al., 2018). It thus may take many centuries for sediments to recover to their original physical and biogeochemical state, which is important for their associated biota (Haffert et al., 2020; Jones et al., 2017; König et al., 2001; Volz et al., 2020; Vonnahme et al., 2020).

Ecological studies following small‐scale experiments that simulated mining operations have shown that whereas densities and diversities of some faunal taxa can recover to or even exceed predisturbance levels, community composition remains affected after decades (Gollner et al., 2017; Jones et al., 2017). Typically within one year there is some recovery in faunal density and diversity for meiofauna and mobile megafauna, with recovery being highly variable among taxonomic groups and locations. There is a general trend that mobile fauna recovers faster than sessile fauna (Gollner et al., 2017; Jones et al., 2017). However, very few faunal groups return to baseline or control conditions after two decades (Gollner et al., 2017; Jones et al., 2017). For example, nematode communities had not recovered to predisturbance diversity 26 years after the disturbance (Miljutin et al., 2011), and sessile epifauna had not recovered after 37 years (Vanreusel et al., 2016). Very little is known about the recovery potential of foraminifera, although they are diverse and abundant in sediments and on nodules (Goineau & Gooday, 2017; Gooday et al., 2015; Lejzerowicz et al., 2021). A recolonization experiment on a nodule‐free deep seabed in ~1500 m depth off Japan showed that defaunated sediments were colonized by most foraminifera species within one year, but population sizes were 50 times smaller. In contrast, the used artificial substrates (glass beads) were only colonized by shallow and opportunistic species in the top fluffy layer that had been deposited upon glass beads (Kitazato, 1995). Recovery of microbial communities at impacted nodule areas is slow, with recent studies showing that microbial abundance, activity, and loop in sediments are still impaired 26 years after impact (de Jonge et al., 2020; Vonnahme et al., 2020).

Removal of nodules may be especially critical for the distinct microbial, foraminiferal, and faunal communities living on and in the nodules. The habitat for communities living in the crevices of porous polymetallic nodules (Pape et al., 2021; Thiel et al., 1993) will be lost. The faunal composition in the crevices differs from that in the sediment surrounding the nodules (Thiel et al., 1993), but crevice fauna is to our current knowledge not endemic to the nodule crevices and only adds little to the total meiobenthic abundance and diversity (Pape et al., 2021). Microbial and foraminiferal communities in crevices have to our knowledge not yet been studied. Microbial communities associated with polymetallic nodules are distinct from those in the sediments, and enriched by metal‐cycling and nitrifying microbes (Molari et al., 2020; Shulse et al., 2017). According to Gooday et al. (2015), foraminiferal communities on nodules are characterized by a high number of morphospecies (they found 75 morphospecies on seven nodules), that are widely distributed at regional scales in the abyssal Pacific but not necessarily at global scales. Nevertheless, many morphospecies are too rare to provide information on their geographical distributions (Gooday et al., 2021). Furthermore, polymetallic nodules harbor metazoan communities including, for example, corals or sponges that can only live on the hard nodule substrate, but not on the soft abyssal sediments (Amon et al., 2016). Interestingly, the epifauna on hard nodule substrate differs from epifauna on the hard basaltic substrates of seamounts in the same region, so that seamounts do not provide potential refuge areas for the nodule‐associated fauna (Cuvelier et al., 2020). Stalked sponges on nodules were identified as key structural species, supporting a high diversity of associated fauna and playing critical roles in the food web (Purser et al., 2016; Stratmann et al., 2021).

Nodules represent the dominant topographic structure within abyssal plains, influencing local, small‐scale hydrodynamic regimes. In turn, they affect food availability and overall habitat heterogeneity, which are important structuring factors for benthic communities. As a result, the loss of nodules, but also the modification and loss of sediments may cause benthic community shifts that may persist over geological time scales at mined sites (Gollner et al., 2017).

In areas beyond national jurisdiction, the ISA is mandated under the *UN Convention on the Law of the Sea* to organize, regulate, and control all mineral‐related activities for the benefit of mankind as a whole, ensuring the effective protection of the marine environment from harmful effects that may arise from deep‐seabed‐related activities. Permission to conduct seabed mining may not be granted “where substantial evidence indicates the risk of serious harm to the marine environment” (1982 UN Convention on the Law of the Sea, Art. 162(2)). The need for establishing mitigation measures to reduce impacts to the point at which serious harm to the marine environment does not occur is recognized by the ISA. On land and in coastal areas, environmental risks are typically managed through the application of the mitigation hierarchy (avoidance, minimization, rehabilitation and/or restoration, offset; see Table 1). In the CCZ, the ISA has for example established a Regional Environmental Management Plan (CCZ‐EMP) that sets aside large Areas of Particular Environmental Interest (APEIs), which are protected from future exploitation of mineral resources (Figure 1). There are efforts by industry‐academia consortia to develop mining vehicles that minimize the environmental footprint as much as is reasonably possible (e.g., www.blueharvesting-project.eu). Furthermore, the ISA “draft exploitation regulations” include a section on the Environmental Management and Monitoring Plan, which shall contain details of any practicable restoration of the project area (ISBA, 2019).

**Table 1:** The mitigation hierarchy (BBOP, 2012; BBOP & UNEP, 2010; IFC, 2019)

Step 1	Avoidance: action(s) that avoid impacts that cause significant loss in biodiversity and associated ecosystem services.
Step 2	Minimization: action(s) that are taken to minimize impacts that cannot be avoided (reduce duration, intensity, and extent).
Step 3	Rehabilitation and/or restoration: action(s) taken to assist the recovery of ecosystems that have been degraded, damaged, or destroyed. Rehabilitation emphasizes the reparation of ecosystem processes, productivity, and services. The restoration target is to establish a self‐supporting habitat similar to the “original” habitat prior to impacts, including re‐establishment of the pre‐existing biotic integrity in terms of species composition and community structure.
Step 4	Off‐set: measurable conservation outcomes resulting from actions designed to compensate for significant residual adverse biodiversity impacts after appropriate avoidance, minimization, and rehabilitation and/or restoration measures have been taken. The goal is no net loss and preferably a net gain of biodiversity.

In practice, ecological restoration in the deep sea remains understudied, and knowledge on restoration in the deep sea is still in its infancy (Cuvelier et al., 2018; Da Ros et al., 2019; Van Dover et al., 2014). To date, there are no studies on potential restoration actions in nodule areas impacted by mining activities. The Belgian company Global Sea Mineral Resources (GSR), a member of the DEME group, conducted the first small‐scale mining tests with its hydraulic preprototype nodule collector “Patania II” in the Belgian and German contract areas in the CCZ in 2021. Prior to the tests, GSR and the German contract holder “Bundesanstalt für Geowissenschaften und Rohstoffe” (BGR) both published complete Environmental Impact Statements (EIS) related to these mining tests (https://www.isa.org.jm/minerals/environmental-impact-assessments). The environmental impacts of “Patania II” were monitored by these contractors and in addition also independently by European scientists of the research project “MiningImpact2—Environmental Impacts and Risks of Deep‐Sea Mining” (https://miningimpact.geomar.de/miningimpact-2; https://miningimpact.geomar.de/documents/1082101/1317712/JPIO_MiningImpact2_Proposal_public.pdf/1dfbf9fa-d0cc-4905-b668-49aba79a5ebd). As one aspect of the “MiningImpact2” project, we aim to test the feasibility of restoration measures after dredge and epibenthic sledge impact and after a test‐mining activity in the deep sea.

We present two different scientific restoration experiments. In order to test potential restoration measures that could facilitate the recovery of nodule‐associated communities after their removal from the seafloor, we have deployed artificial hard substrates in disturbed areas. We manufactured ceramic artificial nodules made out of clay and deployed them in the BGR and GSR contract areas in the CCZ at 4100–4500 m depth. In the next years and decades, we aim to study the general feasibility of the approach, the role of substrate type for biogeochemistry in sediments below substrates, and for biofilm and biota succession on nodules and in sediments below the substrates. To test potential restoration measures that could facilitate the restoration of sediment communities after compaction of the sediment by mining gear, we “decompacted” the newly exposed, more compact subsurface sediment within a recent epibenthic sledge track by operating a rake with a remotely operated vehicle (ROV). Considering the slow natural recovery rates of deep‐seafloor communities, it is our intention to sample at the decompaction site and to recover these artificial hard substrates about every five years, with an intended project duration of ~30 years.

## METHODS

### Artificial nodules

#### Artificial nodule production

Artificial ceramic nodules, made of clay, were fired in a kiln (oven) at ~800 to 1200 °C. This method was developed to accommodate for: (1) hard substrate as a replacement for polymetallic nodules, (2) natural material use, and (3) using material present in situ at the study sites. In addition, it is conceivable that such artificial nodule production could be linked in the future to a reduction of the return water plume by filtering the seawater separated from nodules produced on ships and using the filtered material (clay and nodule debris) for artificial nodule production on the ship. Artificial nodules may also be generated from on‐ or offshore mining waste.

**Figure 2:**
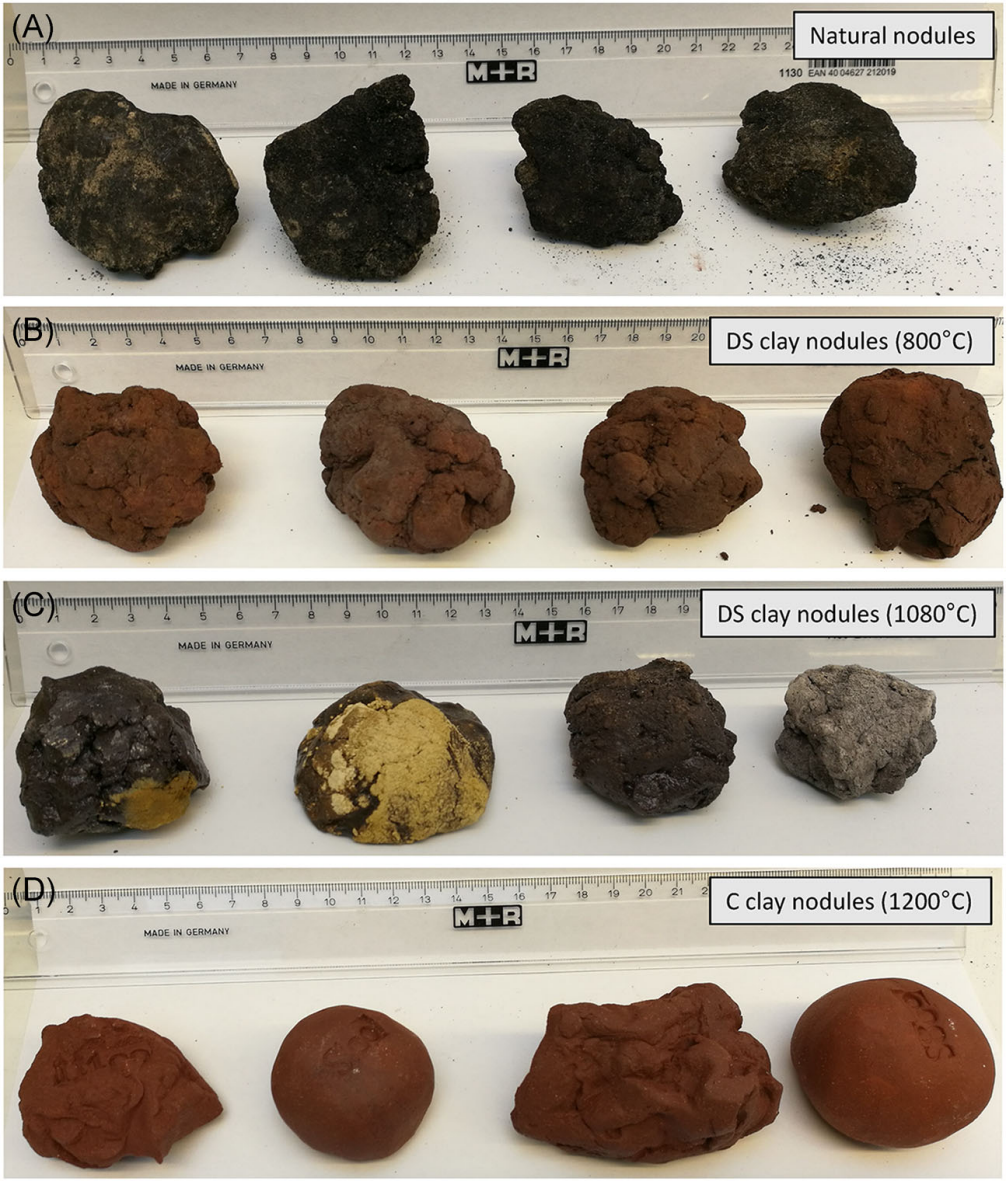
Photographs of (A) natural polymetallic nodules; (B) artificial nodules made from deep‐seafloor (DS) clay and fired in a kiln (oven) at 800 °C, producing a brownish color with a friable surface; (C) artificial nodules made from deep‐seafloor clay and fired at 1080 °C, producing a black color with hard glassy surface, and (D) artificial nodules made from commercial (C) clay (C364) and fired at 1200 °C, producing a reddish and hard surface area

Deep‐seafloor clay was collected from the eastern German contract area in the CCZ (ca. 11°55′N; 116°15′W) by using a boxcorer during expeditions with RV *SONNE* (SO262 and SO268) in 2018 and 2019 and shipped to Europe. The seafloor clay was air‐dried prior to further processing. Clay was weighed into 100 g portions and was formed by hand into a size and shape mimicking the nodules in the German exploration area, covering a sediment area of ~3 × 5 cm and ~3 cm in height. The clay nodules were fired at 800 °C, producing a brownish color with a friable surface, and at 1080 °C, producing a black color with hard glassy surface. Due to the natural variation of mineral composition of the seafloor clay, the resultant nodules had varying colors (Figure 2). In addition to the seafloor clay, we used commercial clay C364 from the German company Goerg and Schneider, weighed it into 100 g portions, formed shapes of ~3 × 5 × 3 cm, and fired them at 1200 °C, a standard temperature used for this clay type in art ceramics, producing a reddish and hard surface area (Figure 2). The same was done for 180 g portions, resulting in ~4 × 5 × 4 cm large artificial nodules. A subset of nodules was put inside a pressure container at Royal NIOZ at 400 bar, to test the resistance of artificial nodules prior to in situ deployment at ~4000 m depth. None of the artificial nodules broke in the pressure container.

#### Mineral content evaluation of artificial and natural nodules

The mineral content of the wet commercial clay C363 is: SiO_2_ (59.55%), Al_2_O_3_ (16.04%), TiO_2_ (0.98%), Fe_2_O_3_ (8.10%), K_2_O (3.64%), Na_2_O (5.21%), CaO (0.90%), MgO (3.04%). The mineral contents of air‐dried seafloor clay, air‐dried commercial clay, fired artificial nodules, and of the natural nodules were evaluated (Supporting Information Appendix S1). Each bulk sample was reduced to a fine powder and front‐side loaded onto polymethyl methacrylate (PMMA) sample holders with a cavity diameter of 25 mm. The samples were run on a Bruker D8 Advance with a *θ*/*θ* goniometer. We used a primary Soller slit of 2.5°, a variable divergence slit, resulting in a constant irradiated length of 20 mm, a motorized anti‐scatter screen, and an anti‐scatter slit of 18 mm. X‐ray powder diffraction patterns were recorded from 3 to 70° 2*θ*, in steps of 0.02°, counting for 0.85 s per step, using Cu*K*α radiation (40 kV/40 mA). Samples were spun continuously (0.25 Hz) during measurement.

#### Frames with artificial and natural nodules

**Figure 3:**
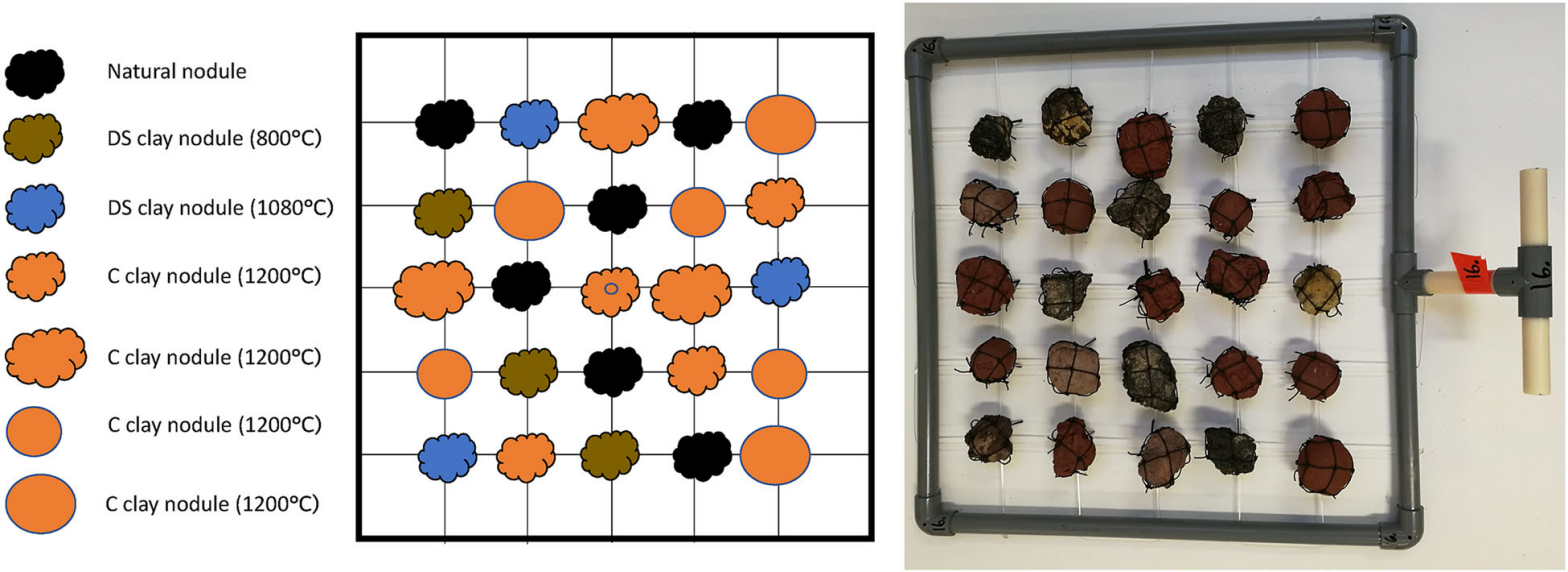
Illustration (left) and photograph (right) of the experimental setup of artificial and natural nodules mounted onto one frame (“nodule frame”). Artificial nodules consist of different clays (clay from the deep seabed or clay for commercial purposes extracted from land), have a different texture according to firing temperatures, have different surface areas and textures (smooth vs. rough), and different sizes (small and large). Natural nodules are used as a scientific control

The amount and size of colonization substrates per frame were chosen based on an average natural nodule in situ conditions in the German test‐mining site. In this area, ~18% of the seabed is covered with nodules, and ~70% of polymetallic nodules are <4 cm in diameter (A. Vink, BGR, personal communication, 2021). For each experimental setup (“frame”), we mounted a total of 25 nodules into a 50 × 50 cm large polyvinyl chloride (PVC) frame (diameter of PVC‐tube: 2 cm) (Figure 3, Table 2). Nodules were fixed with a net (mesh size ~2 × 2 cm) onto a plastic thread (diameter: 2 mm) that was woven into the frame (Figure 3). The colonization substrates in the frame cover ~20% of the seabed below the frame and have sizes and shapes similar to natural nodules. All artificial nodules made from deep‐seafloor clay have a shape mimicking the natural nodules with a rough surface structure. Commercial clay nodules have shapes mimicking the natural nodules, with a rough surface structure, and ball‐like shapes with a smooth surface structure (see Figure 2).

**Table 2:** Number and type of nodules on each frame

Material	Structure	°C	cm^2^	g	#Nodules
Deep‐seafloor clay	Rough	1080	~15	180	3
Deep‐seafloor clay	Rough	800	~15	100	3
Commercial clay	Rough	1200	~15	100	3
Commercial clay	Smooth	1200	~15	100	3
Commercial clay	Rough	1200	~20	180	3
Commercial clay	Smooth	1200	~20	180	3
Commercial clay	Rough & artificial sponge	1200	~15	100	1
Natural nodule	Natural nodule (rough)		~15		6

*Note*: Artificial nodules were produced from different materials (deep‐seafloor clay or commercial clay), with rough or smooth surface structure, and large or small size (covered sediment area: ~15 or 20 cm^2^). Sizes varied slightly but the mass of commercial clay was set at 100 g prior to firing for small nodules (resulting in 3 × 5 × 3 cm size) and at 180 g prior to firing for large nodules (resulting in 4 × 5 × 4 cm size). Deep‐seafloor clay was preweighed into 100 g portions for 800 °C firing processes, and into 180 g portions for 1080 °C, both producing final sizes of ~3 × 5 × 3 cm). In addition, a total of 11 frames was equipped with an artificial sponge that was mounted onto one commercial‐clay nodule.

Each frame contained a total of three small rough deep‐seafloor clay nodules fired at 800 °C, three small rough deep‐seafloor clay nodules fired at 1080 °C, three small rough commercial clay nodules (fired at 1200 °C), three small smooth commercial clay nodules (fired at 1200 °C), three large rough commercial clay nodules (fired at 1200 °C), three large smooth commercial clay nodules (fired at 1200 °C), and one small smooth commercial clay nodule (fired at 1200 °C) with an attached artificial sponge (Table 2, Figure 3). Typically, three frames per area were equipped with a single artificial plastic kitchen sponge, mimicking natural sponges. All frames were equipped with six natural nodules as controls. Natural nodules were exposed to air for more than a year before they were mounted on the frames and thus were free of alive marine taxa. Staining of alive specimens after recovery will be used to discriminate between any possible pre‐ and postsettled fauna on natural nodules.

#### Deployment of nodule frames

In 2019, during the research cruise SO268 of the project MiningImpact2 (MI2), a total of 86 frames, equipped with a total number of 2150 nodules (25 per frame), were deployed by the ROV KIEL 6000 in different locations within the German contract area (Table 3, Figures 5 and 6). Three frames were deployed at a site within the MI2 trial area before impact, 26 frames were deployed at a site within the MI2 reference area, 27 at a no‐nodule site, and 27 at a site within the MI2 dredge area, where a 1‐m wide chain dredge was towed 11 times over the seafloor in E‐W direction to cause disturbance in an attempt to mimic test mining a few days prior to the deployment. In addition, three frames were positioned at the decompaction site, which was artificially “decompacted” after disturbance with an epibenthic sledge (see text below). The original plan to deploy all frames in 2019 could not be carried out since the DEME‐GSR mining test had to be postponed to 2021 due to technical problems with the prototype vehicle's power and communications cable.

**Table 3:** Deployment of nodule frames

Station number	Date deployment (UTC)	ROV Dive #	Latitude	Longitude	Contract holder	Site	Depth (m)	# Frames	# Nodules	Recovery
SO268/1_026_1	05.03.2019 20:36	3	11°55.719′N	117°1.456′W	BGR/GER	MI2 trial site (before Patania II impact)	4088	3	75	28.04.2019
SO268/1_035_1	08.03.2019 21:20	6	11°50.646′N	117° 3.554′W	BGR/GER	MI2 reference site	4136	26	650 (2)	tbd
SO268/2_158‐1	26.04.2019 20:18	24	11°51.011′N	117°23.033′W	BGR/GER	No‐nodule site (naturally)	4277	27	675 (3)	tbd
SO268/2_188_1	12.05.2019 15:23	29	11°51.617′N	117°00.747′W	BGR/GER	MI2 decompaction experiment site	4127	3	75	tbd
SO268/2_188	12.05.2019 18:08	29	11°51.807'N	117°00.694'W	BGR/GER	MI2 dredge site	4125	14	350 (3)	tbd
SO268/2_197	14.05.2019 16:51	31	11°51.862'N	117°00.779'W	BGR/GER	MI2 dredge site	4126	13	325	tbd
IP21‐075ROV1	9.05.2021 09:45	HD14‐190	11°55.783'N	117°01.503′W	BGR/GER	MI2 trial site (after Patania II impact)	4089	15	375 (3)	tbd
IP21‐044ROV1	28.04.2021	HD14‐183	14°06.676′N	125°52.379′W	GSR/BEL	MI2 trial site (after Patania II impact)	4497	15	375	tbd

*Note*: Station number of expedition, date, and start‐time of deployment, ROV dive number (#), latitude, longitude, exploration contract holder/Sponsoring State (Bundesanstalt fuer Geowissenschaften und Rohstoffe/Germany; Global Sea Mineral Resources/Belgium), site name (MI2 = MiningImpact2), water depth, number of frames deployed per site, and number of nodules deployed. Number of nodules per frame is 25, of which 19 are artificial nodules and six are natural nodules as control. In parentheses number of nodules equipped with an artificial sponge (11 nodules on a total of 11 frames). Three nodule frames were recovered after 8 weeks, all other nodule frames shall be recovered in the next years and decades.

Abbreviation: tbd, to be determined.

In 2021, a total of 30 frames, equipped with a total number of 750 nodules (25 per frame), were deployed during the BGR research cruise MANGAN 2021. They were deployed using the ROV HD14 onboard the MV ISLAND PRIDE a few days after GSR's successful mining tests with Patania II. Fifteen frames were deployed in the GSR contract area and 15 frames were deployed in the BGR contract area in ~4500 and ~4100 m depth, respectively (Table 3, Figures 5 and 6).

### Sediment decompaction

#### Physical decompaction of sediment

**Figure 4:**
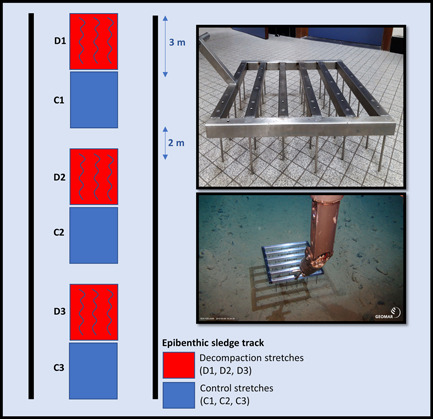
Photograph of the rake used to decompact the sediment (top right), photograph showing in situ decompaction of the sediment (bottom right), and illustration of epibenthic sledge track showing decompaction patches and control patches (left)

To decompact the sediment, a 50 cm wide and 50 cm long stainless‐steel frame was constructed, with six bars containing alternating rows of five or six metal pins (10 cm height), resulting in a “decompaction rake” covering a total surface area of 0.25 m^2^ (Figure 4).

**Figure 5:**
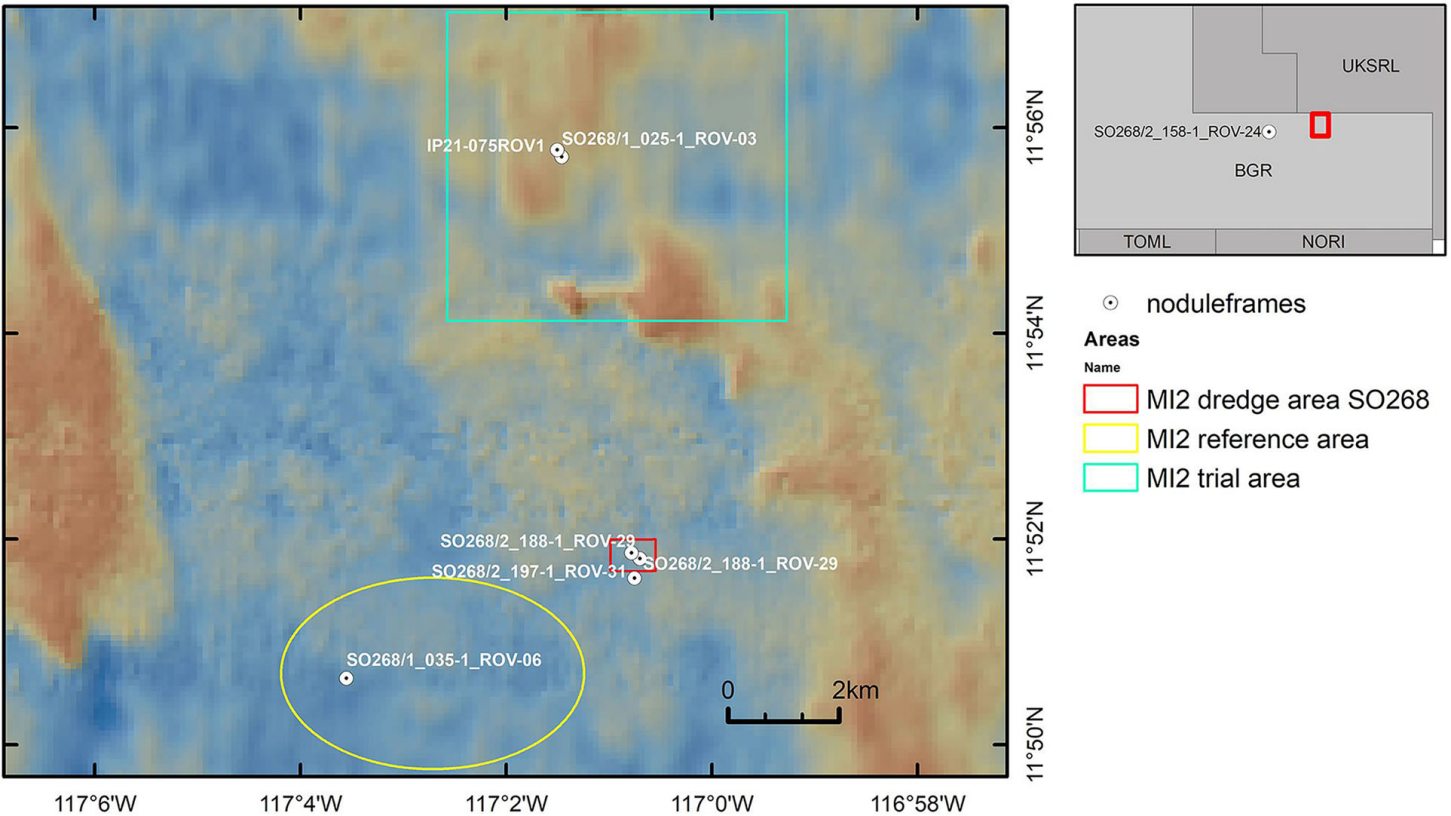
Map with deployment locations of frames with artificial and natural nodules (nodule frames) in the German contract area. The inset map shows the main areas investigated during the MI2 project as a red rectangle, and also shows the deployment location of nodule frames in an area devoid of natural nodules (no‐nodule site). Inside the main area investigated during the MI2 project, nodule frames were deployed at sites (indicated by white/black dots) within the MI2 trial area after test‐mining by Patania II, within the MI2 dredge area, and within the MI2 reference area. The ROV dive numbers of deployments are given. Recovery of nodule frames is planned in the next years and decades

**Figure 6:**
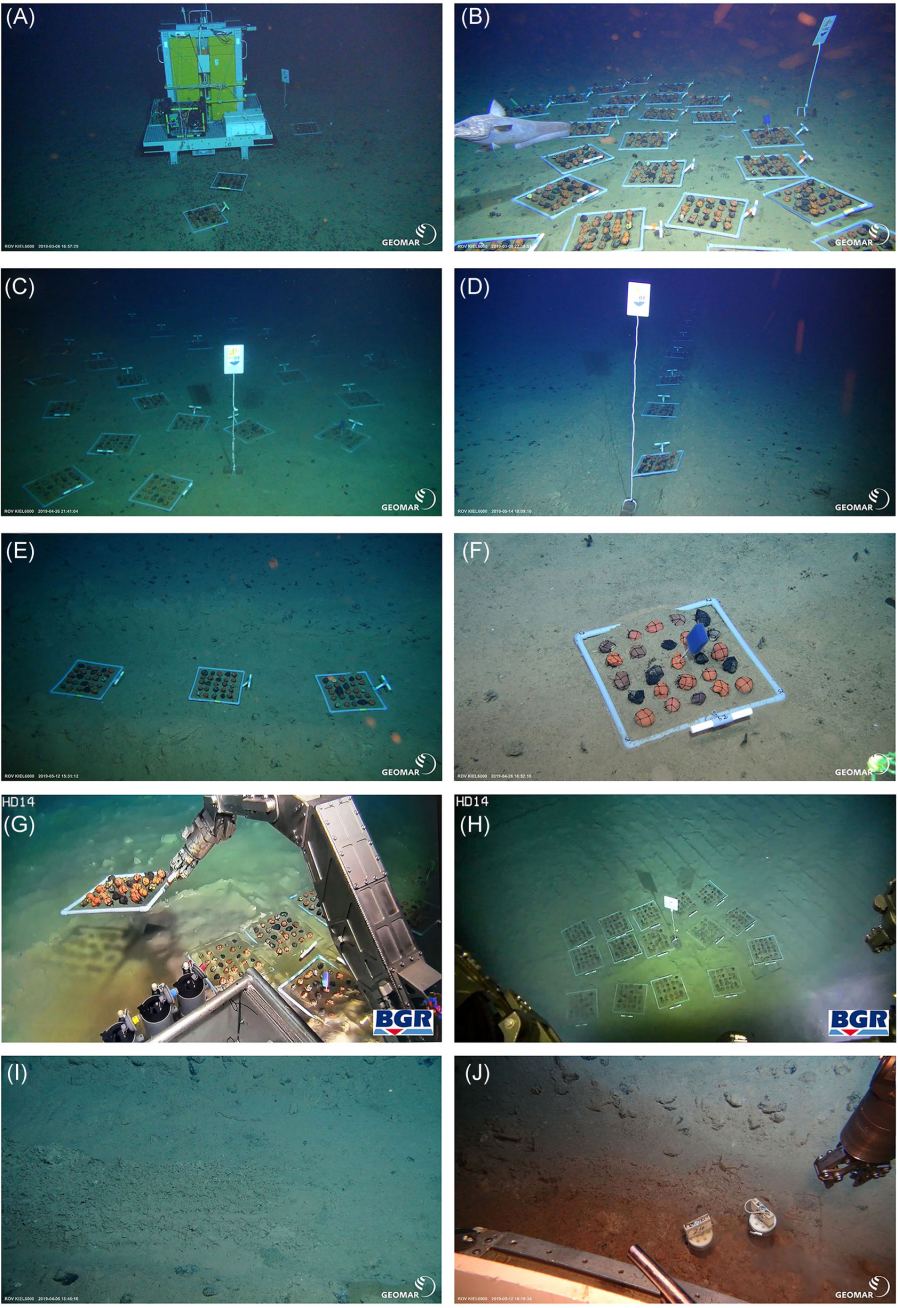
Photographs of scientific restoration experiments in disturbed polymetallic nodule areas in the Clarion‐Clipperton Zone in ~4100 and ~4500 m depth. (A–H) Deployed nodule frames with ceramic artificial nodules and natural nodules as control. (A) MI2 trial site before test‐mining (BGR/GER), (B) MI2 reference site (BGR/GER), (C) no‐nodule site (BGR/GER), (D) MI2 dredge site (BGR/GER), (E) MI2 epibenthic sledge track after decompaction (BGR/GER), (F) close‐up of a nodule frame with an artificial sponge, (G) MI2 trial site after Patania II test‐mining impact in the BGR/GER area, (H) MI2 trial site after Patania II test‐mining impact in the GSR/BEL area. (I–J) Decompacted sediment. (I) Decompacted sediment patch in the MI2 decompaction experiment site (BGR/GER), and (J) push core sampling in one of the decompacted sediment patches

On 6 April 2019, the decompaction rake was manipulated by the ROV KIEL 6000 arm to make three decompaction patches inside an epibenthic sledge track in the German contract area that had been towed during SO239 in 2015 (Station SO239_024_EBS) (Figure 6I,J). Each decompacted patch was 3 m long and the distance between two consecutive patches was roughly 2 m (Figure 4). The MI2 decompaction experiment site was marked to allow for long‐term monitoring of the potential effect of decompaction on environmental variables (granulometry and nutrients) and faunal communities.

#### Push‐core sampling in the decompaction area and sample processing

As a first sampling action, the decompaction experiment site was revisited with the ROV KIEL 6000 to collect sediment samples from the disturbed area, including the decompacted patches and none decompacted patches by means of push core (PUC) sampling on 12 May 2019 (Figures 4 and 6J). Each patch was sampled by two PUCs, one for environmental variables (granulometry and nutrients) and one for meiofauna community analyses (Supporting Information Appendix S2). All PUCs were sliced per centimeter down to 5 cm depth and stored either frozen (i.e., −80 °C; environmental variables) or in a buffered 4% formaldehyde solution (for community analyses) for further analysis at Ghent University. Statistical analysis was performed for the environmental variables (granulometry and nutrients) to test for spatial differences between samples taken within and outside the decompacted patches. This was achieved through a one‐way ANOVA (factor: sediment type) to assess differences between sediment types in terms of bulk (0–5 cm) averages, in combination with a two‐way ANOVA (factors: sediment types, depth layer) to assess these differences for every depth layer (0–1, 1–2, 2–3, 3–4, 4–5 cm). Univariate analyses and visualization of the results (bar plots) were performed in R Studio (version 3.6.1).

## RESULTS

### Mineral composition of artificial and real nodules

The commercial clay sample is different in composition from the seafloor clay: the former is dominated by the detrital products of chemical weathering of previously existing rocks, whereas the latter consists of a mixture of sedimentary minerals typical of a pelagic clay and mafic igneous minerals typical of an oceanic basalt (Supporting Information Appendix S1). The commercial clay sample consists of illite/muscovite and kaolinite. In addition, it contains quartz, potassium feldspar, hematite, goethite (minor), and rutile (trace). The deep‐sea clay consists of quartz, hematite, zeolite (phillipsite), plagioclase (labradorite), monoclinic pyroxene (augite‐diopside), and halite.

The composition of the artificial nodules differs from that of the commercial or seafloor clay from which they were made in that neoformed minerals appear at the expense of some of the previously existing minerals. In the artificial nodule made from commercial clay, the neoformed minerals cristobalite (minor) and mullite appeared, the hematite peaks became stronger, the quartz peaks weaker, and the illite/muscovite, kaolinite, and potassium feldspar peaks disappeared. Mullite, though rare in rocks, is normally present in porcelain as it is produced by firing noncalcareous kaolinitic clay. The artificial nodule made from seafloor clay and fired at lower temperature has a composition similar to the dried deep‐sea clay. The artificial nodules fired at high temperatures contain hematite, cristobalite, potassium feldspar (anorthoclase), quartz, and spinel. The hematite peaks became stronger, the quartz peaks weaker, and the halite peaks disappeared. The composition of the artificial nodules fired at high temperature thus reflects the formation of firing products above 1000 °C, rather than the original mineralogy.

The natural nodules consist of turbostratically disordered vernadites (phyllomanganates) with hexagonal symmetry (Wegorzewski et al., 2015). In addition, feroxyhyte, quartz, montmorillonite, and phillipsite were detected. There is no qualitative difference in mineralogical composition between the four analyzed deep‐sea nodule samples—that is, between the inside and the outside layers of the nodule or between the upper and lower sides of the nodule.

Artificial and natural nodules are, thus, very different in terms of composition: the artificial nodules produced in this study were dominated by silicates, whereas the seafloor nodules are dominated by manganese and iron oxides (Supporting Information Appendix S1).

### Recovery of nodule frames

The three frames that were deployed at the MI2 trial site on 5 March 2019 were recovered eight weeks later with the ROV KIEL 6000 on 28 April 2019, with the main purpose of testing whether frames with nodules can be successfully deployed and recovered without physical damage. Recovery of frames by ROV was successful and each frame was put into a single biobox. Of these recovered frames, only one carried one mobile polychaete individual. However, due to technical problems, the water of the bioboxes was not sieved, thus an abundance of mobile fauna may be underestimated. No sessile epifauna had settled after eight weeks.

All other frames should be recovered (~5 frames per area every ~5 years) within the next years and decades.

### Sediment decompaction

#### Granulometry and nutrients one month after sediment decompaction

In the upper layers (i.e., 0–1, 1–2 cm) of the sediment, sand fractions are proportionally lower within the decompacted samples, whereas TOC values are higher (Supporting Information Appendix S3). Statistical testing (i.e., one‐way ANOVA, factor: sediment type) for the bulk (0–5 cm) averages confirms the lower average sand fraction (*p* = 0.0414) for the decompacted samples (13.99 ± 1.79) compared to the control samples (14.89 ± 2.34). However, there were no significant differences among any of the environmental variables, including granulometry, when a two‐way ANOVA (factors: sediment type × depth layer) was performed.

## DISCUSSION

### Production of artificial nodules

The production and deployment of artificial nodules were successful, as none of the ceramic artificial nodules imploded during transport to ~4100 and ~4500 m depth. Future recoveries will reveal if there is a difference in artificial nodule stability among the chosen firing temperatures for deep‐seafloor clay nodules. In general, we expect that there will be no such effect, as ceramics typically show little sign of degradation after hundreds of years of seawater exposure. Ancient ceramics found in old shipwrecks are of good ceramic quality (Heng, 2019). For future production, it should be noted that the firing process of the deep‐seafloor clay nodules produced potentially toxic gases and the natural salt content in the seafloor clay caused erosion in the electric oven. Firing in a gas oven avoids the problem of erosion.

### Does the type of artificial nodule influence recruitment?

In our experimental set‐up, we have selected different substrates with varying mineral content to test if this may influence the settlement of deep‐sea organisms. In addition, we used different surface structures (smooth vs. rough) to investigate if enhanced surface structure on rough nodules may enhance abundance and influence the composition of the communities of recruited organisms. We used different nodule sizes, to investigate if nodule size and height affect recovery. None of the artificial nodules used in this study mimics the high porosity of natural nodules (Hein et al., 2020), and would not be able to aid restoration of nodule crevice fauna (Pape et al., 2021; Thiel et al., 1993).

A few experiments using nodules in shallower and more productive waters provided first insights into factors that may influence community composition on natural and artificial nodules at 4100 m depth. On natural nodules, significant differences in faunal abundance and species composition were detected on smooth and rough nodules, and at the summit and base of the nodules, which may be related to larval responses to surface structure and microcurrents (Mullineaux, 1989). Boundary shear stress and benthic flux of particles (i.e., food or larvae) may increase from nodule base to summit, while particle contact rate and deposition decrease (Mullineaux, 1989). To test the effect of elevation, Mullineaux (1988) built a frame with a lower tier containing nodules and ceramic substrates and an upper tier (20 cm elevation from the ground) with nodules. She deployed the frames in a “no‐nodule” area in ~1200 m depth in the Santa Catalina Basin, about 30 km offshore, and recovered them after seven weeks and after two years. She observed that organism abundance increased with time (i.e., from ~<1 individual/cm^2^ to ~2–5 individual/cm^2^), that elevation of nodules influenced community structure, and that taxon richness was higher on nodules than on ceramics (Mullineaux, 1988). A settlement experiment, using plates that had been deployed at Cross seamount in 400 m depth, also demonstrated that larval settlement may be a function of very small‐scale variations in the boundary layer flow, reflecting, for example, larval supply to the plate, larval retention on the plate surface, and active larval responses to the flow regime over the plates. In addition, several taxa were recruited exclusively onto plates covered with a finely powdered ferro‐manganese crust, suggesting active selection for substratum composition or texture (Mullineaux & Butman, 1990).

Colonization experiments using hard substrate islands in sedimented surroundings, as well as biofouling studies can provide further insight into the potential suitability of artificial nodules for restoration. Typically, colonization is influenced by substrate type (Bellou et al., 2011), with artificial substrates colonized only by a subset of taxa found in natural communities (Beaulieu, 2001). A colonization experiment in a deep‐sea, long‐term observatory in the Arctic Ocean (HAUSGARTEN) showed that most common recruiters on hard substrate panels were opportunistic, while the main natural structuring species (hexactinellid sponges) had not colonized, suggesting that community succession in the Arctic takes longer than the two decades spanned by the study (Meyer‐Kaiser et al., 2019).

To date, it is uncertain whether artificial nodules are suited to host communities in a similar way to natural nodules, and thus can be effective for restoration. Experiments by Mullineaux in “no‐nodule” areas suggest that there may be active selection for substratum composition and that microcurrents associated with nodule size, nodule shape, and elevation may influence faunal recruitment and species composition. Our observation of no sessile epifauna and only a single mobile polychaete on one of the three frames recovered after one month provides an initial indication that recruitment on abyssal plains may be slower than in shallower areas that are closer to the coast and more productive. Only future recoveries of artificial nodules in the next years and ~3 decades will reveal whether parameters such as mineral content, surface structure, or size influence the recruitment of organisms on nodules in the CCZ.

### Nodules and their influence on sediments

Baseline knowledge on the effect of nodules on the biogeochemistry of sediments, local hydrodynamics (including microdistribution of particles like food and larvae), and fauna living in and on sediments is extremely scarce. Mullineaux (1989) found that boundary shear stress and benthic flux of particles may increase from nodule base to summit, while particle contact rate and deposition decrease (Mullineaux, 1989). Hence, nodule form will have an impact on the distribution of particles (food and larvae) in sediments as well. In the Peru Basin, solid‐phase and porewater analyses of sediments were carried out in an area in which nodules had been plowed under during a DISturbance and reCOLonization experiment (DISCOL) in 1989. Results demonstrated that 26 years after disturbance, predisturbance porewater conditions had been re‐established, but the upper sediment layer indicated substantial differences in metal distribution (Paul et al., 2018). To date, it is unknown whether and how biodiversity in sediments is affected by nodules laying on top and within the sediments. Comparison of microbial communities of the nodules with those inhabiting the sediments suggests that microbes associated with the nodules may have an important role not only in metal‐cycling but also in carbon and nitrogen cycles (Molari et al., 2020). Furthermore, differences in nodule community composition between the CCZ and the Peru Basin suggest that changes in environmental settings (i.e., sedimentation rates, nodule features) also play a significant role in structuring the nodule microbiome (Molari et al., 2020). Yet, spatial turnover and metabolic activities, and factors and processes controlling nodule colonization are largely unknown. We urge that such baseline knowledge needs to be collected in the future in order to better understand the impacts of nodule mining, to assess the success of restoration measures, and to take this information into account for any potential future restoration action.

### Decompacted sediment

In addition to the removal of polymetallic nodules, the mining vehicle will also cause modification of the soft sediment from which the nodules are collected. The mining‐vehicle by its weight on the seafloor may compact the sediment. Quantities of sediment will be displaced, most likely 5–15 cm of the upper sediment layer, thereby exposing deeper and denser sediments that are depleted in organic matter. In addition, we expect a return of some of the extracted sediments and the creation of a sediment plume that will resettle onto the fresh collector tracks. The composition and density of this sediment layer are currently unknown. These changes in sediment composition and the resulting shifts in biogeochemical processes will likely affect benthic recolonization after mining (Christiansen et al., 2020; Levin et al., 2016).

Our experiment was conducted in a four‐year‐old epibenthic sledge track in the German contract area. Whereas the removal of surface sediments is well mimicked by these tracks, the intensity of disturbance during mining will be strongly dependent on the technology used (i.e., size, weight, and buoyancy of mining vehicle and the type of collection technology used, in turn influencing the level of vertical penetration). Therefore, the findings of this experiment will only provide insights into recovery patterns related to sediment decompaction.

Previous studies that investigated trawling impacts on benthic communities and sediment characteristics were mostly conducted at shallower depths (i.e., 400–1500 m) within the framework of deep‐sea fisheries and related activities. The majority of results do, however, compare to mining‐related effects, such as erosion of the upper sediment layers and exposure of denser and nutrient‐impoverished sediments (Clark et al., 2015; Martín et al., 2014). Vonnahme et al. (2020) studied plow tracks after disturbance of nodule fields in the Peru basin (DISCOL) and found clear evidence that benthic organic matter remineralization rates and microbial activities were still reduced after 26 years, especially where the active surface layer was removed and subsurface sediments became exposed. Preliminary findings from our experiment indicate that sand fractions are proportionally lower within decompacted samples, whereas TOC values and carbon/nitrogen (C/N) ratio are slightly higher. Although these results confirm the potential for this restoration action to a certain degree, some limitations must be considered. Due to the limited sampling time, the total number of replicates for both sediment types was reduced to only two PUCs per sediment patch. Additionally, no reference samples were collected from adjacent, undisturbed patches, which makes it difficult to make any robust conclusions. We therefore advise that these shortcomings should be taken into account in future follow‐up experiments at this site.

Community succession following a disturbance in deep‐sea soft sediment is considered to be complex and often unpredictable, but we do know that colonization rates are generally slow (Smith & Hessler, 1987). Experiments on recolonization of azoic sediments, designed to estimate the growth rate and maturity level of species, suggested that recruitment rates, growth, and natural mortality are very low in the deep sea. For example, an 8.4 mm long deep‐sea bivalve was estimated to be 100 years old (Grassle, 1977). Food availability seems to be a determining factor for successful recolonization, as shown by experiments adding variations in nutrition to azoic sediments (Freese et al., 2012; Grassle & Morse‐Porteous, 1987). Thus, enhanced TOC and C/N in upper sediment layers as detected in our decompaction experiment may aid recovery after disturbance.

### Expected outcomes and outlook

The deep sea may be a new frontier for ecological restoration, but it is still in its infancy (Da Ros et al., 2019). Although there is a steady increase in baseline information deriving from the CCZ, natural ecosystem characteristics such as very low productivity, extremely high diversity with only a few dominant species, slow growth rates, and very slow recovery (Bonifácio et al., 2020; Christodoulou et al., 2020; Gollner et al., 2017; Jones et al., 2017; Smith et al., 2008, 2020) all make it extremely challenging to establish an appropriate baseline and reference condition for restoration, or to determine effective potential restoration actions. In addition, restoration in the deep sea may be ~3 to 4 times more expensive than for shallow water ecosystems (Van Dover et al., 2014), and estimates for nodule restoration are at ~22.5 billion USD to produce artificial nodules required to restore the hard habitat of ~15 000 km^2^ (assuming 15 nodules/m^2^, 0.1 UDS per nodule) (Da Ros et al., 2019). Future regulation for the exploitation of deep‐sea nodules should consider the degradation of the ecosystem (removal of nodules), and any associated long‐term or permanent loss of ecosystem services that may have negative environmental and economic consequences. Restoration actions should be part of impact statements, and management strategies should assess the costs and benefits of deep‐sea restoration. A robust scientific assessment will be needed to assess whether the restoration action leads to significant restoration success and thus may be suitable.

Our experiments will help to assess the significance of mining impacts and to understand adverse long‐term effects of nodule removal, providing an evidence base for setting criteria for the definition of “serious harm” to the environment. In case “serious harm” occurs, this can trigger the possibility to set areas aside where mining is not allowed, to deny new applications for seabed exploitation, to suspend or alter or terminate operations, and to hold the contractor and its Sponsoring State liable for any environmental harm it has caused (UNCLOS Art. 162((2) (w) and (x) and 165 (2)(k) and (l) and Annex III Article 18) (Levin et al., 2016). Deep seabed mining of polymetallic nodules will remove the hard substrate and the associated obligate nodule communities at the mined site, and will cause significant adverse change for long periods of time (greater than millennia), as nodule obligate communities cannot recover in nodule‐free habitats (Gollner et al., 2017; Vanreusel et al., 2016). The spatial scale of this significant adverse change is not discussed here, but will depend on the size and geometry of mined areas as well as on species, population, community, and ecosystem characteristics, such as species distribution ranges of nodule obligate fauna and their connectivity to nearby nonmined areas (e.g., risk of source population loss, extinction), or alternation of key linkages between nodule and sediment communities (e.g., change in population density and community structure of fauna that can live on nodules and in sediments; change of food webs; change of biomass production, nutrient recycling and carbon burial). With our experiments, we aim to answer whether and to which degree restoration can reduce adverse changes and “serious harm” caused by nodule mining. Key questions include: Can ceramic nodules act as a substitute material for the nodule communities? Do ceramic nodules have a similar effect on abiotic and biotic sediment characteristics as natural nodules? Can sediment decompaction enhance the recovery of sediment communities? Key challenges to be able to determine restoration “success” include the expected slow recovery rates (long timelines required) and the need for detailed characterization of baseline conditions.

## CONCLUSION

We developed two experimental studies to test the feasibility of (i) artificial nodule placement and (ii) sediment decompaction as restoration actions after disturbing polymetallic nodule fields in the CCZ by dredging and test mining. Considering the slow natural recovery in this deep‐sea ecosystem, these experiments represent prolonged studies for which we expect to gain insights into the succession of nodule communities and the influence of nodules and sediment decompaction on the recovery of communities in the sediments throughout the next 30 years. Our experiments will help to understand the long‐term adverse effects of nodule removal and will provide an evidence base for setting criteria for the definition of “serious harm” to the environment. Due to the long time periods involved, the paucity of baseline information, and the high costs associated with deep‐sea research, robust assessments of the restoration potential of any artificial substrates will take years to decades to achieve. We urge that potential restoration actions such as those proposed in this study should never be seen as a “license to trash,” but as one potential step within the mitigation hierarchy. Nevertheless, this type of research is important and should be included as an adaptive management tool to support conservation strategies within the deep sea.

## CONFLICT OF INTEREST

The authors declare that there are no conflicts of interest.

## Supporting information

This article includes online‐only Supporting Information.

Three  appendices with information on (1) figures on the mineral content of artificial and natural nodules, (2) table on push‐core samples collected in the decompaction area, and (3) figures on environmental variables measured in the decompaction area.

## Data Availability

All data are presented in the manuscript and its supplementary material.
