# Supplementary material for: Restoration experiments in polymetallic nodule areas
Source: Integr Environ Assess Manag. 2021 Nov 10;18(3):682–96. doi: 10.1002/ieam.4541 (PMC9299087; doi:10.1002/ieam.4541)

Supplementary Material

Restoration experiments in polymetallic nodule areas

Integrated Environmental Assessment and Management

Article DOI: 10.1002/ieam.4541

Sabine Gollner*[1], Matthias Haeckel[2], Felix Janssen[3,4], Nene Lefaible[5], Massimiliano Molari[4], Stavroula Papadopoulou[6], Gert-Jan Reichart[1,7], João Trabucho Alexandre[7], Annemiek Vink[8], Ann Vanreusel[5]

[1]Department of Ocean Systems, Royal Netherlands Institute for Sea Research (NIOZ), Den Burg, the Netherlands
[2]GEOMAR Helmholtz Center for Ocean Research Kiel, Kiel, Germany
[3] HGF MPG Joint Research Group for Deep Sea Ecology and Technology, Alfred Wegener Institute Helmholtz Centre for Polar and Marine Research (AWI), Bremerhaven, Germany
[4]HGF MPG Joint Research Group for Deep Sea Ecology and Technology, Max Planck Institute for Marine Microbiology (MPI), Germany
[5]Marine Biology Research Group, Ghent University, Ghent, Belgium
[6]WerkStaat, Heidelberg, Germany
[7]Department of Earth Sciences, Utrecht University, Utrecht, the Netherlands
[8]Federal Institute for Geosciences and Natural Resources (BGR), Hannover, Germany

Appendix 1. Summary of the mineralogical composition and bulk powder X-ray diffractometry results of substrates. Substrates included dried (~20°C) and fired (>800°C) commercial and deep-seabed clay used for the production of artificial nodules, as well as natural nodules for control. X-ray diffractograms show values along the x-axis in degrees 2θ and values along the y-axis in counts per second (CPS). Artificial and natural nodules are very different in terms of composition: the artificial nodules produced in this study are dominated by silicates, whereas the deep-sea nodules are dominated by manganese and iron oxides.

Substrate	°C	Mineral content
Commercial clay	20	Illite/muscovite, kaolinite, quartz, K feldspar, hematite, goethite, rutile (trace)
Deep-seabed clay	20	Quartz, hematite, zeolite (phillipsite), plagioclase (labradorite), clinopyroxene (augite-diopside), halite
Commercial clay	1080	Quartz, hematite, mullite, cristobalite (minor)
Deep-seabed clay	800	Quartz, hematite, plagioclase (labradorite), spinel, halite
Deep-seabed clay	1200	Hematite, anorthoclase, spinel, cristobalite, quartz
Natural nodule	n.a.	10 and 7 Å vernadite, feroxyhyte?, quartz, smectite (montmorillonite), zeolite (phillipsite)

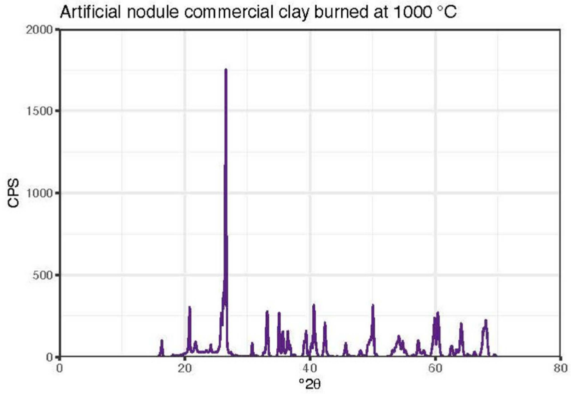

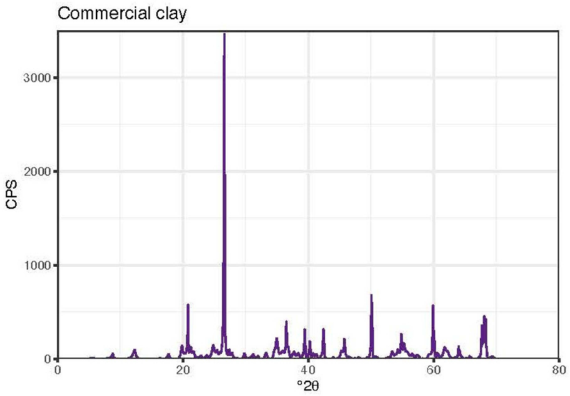

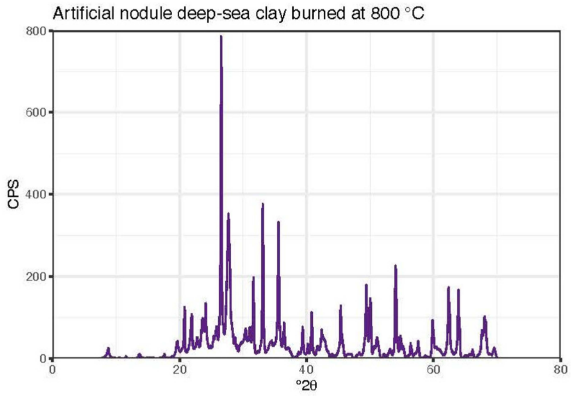

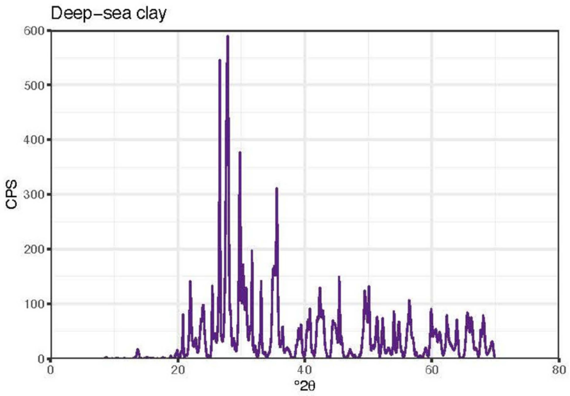

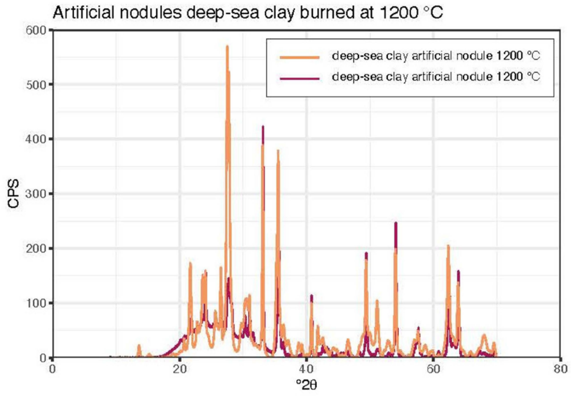

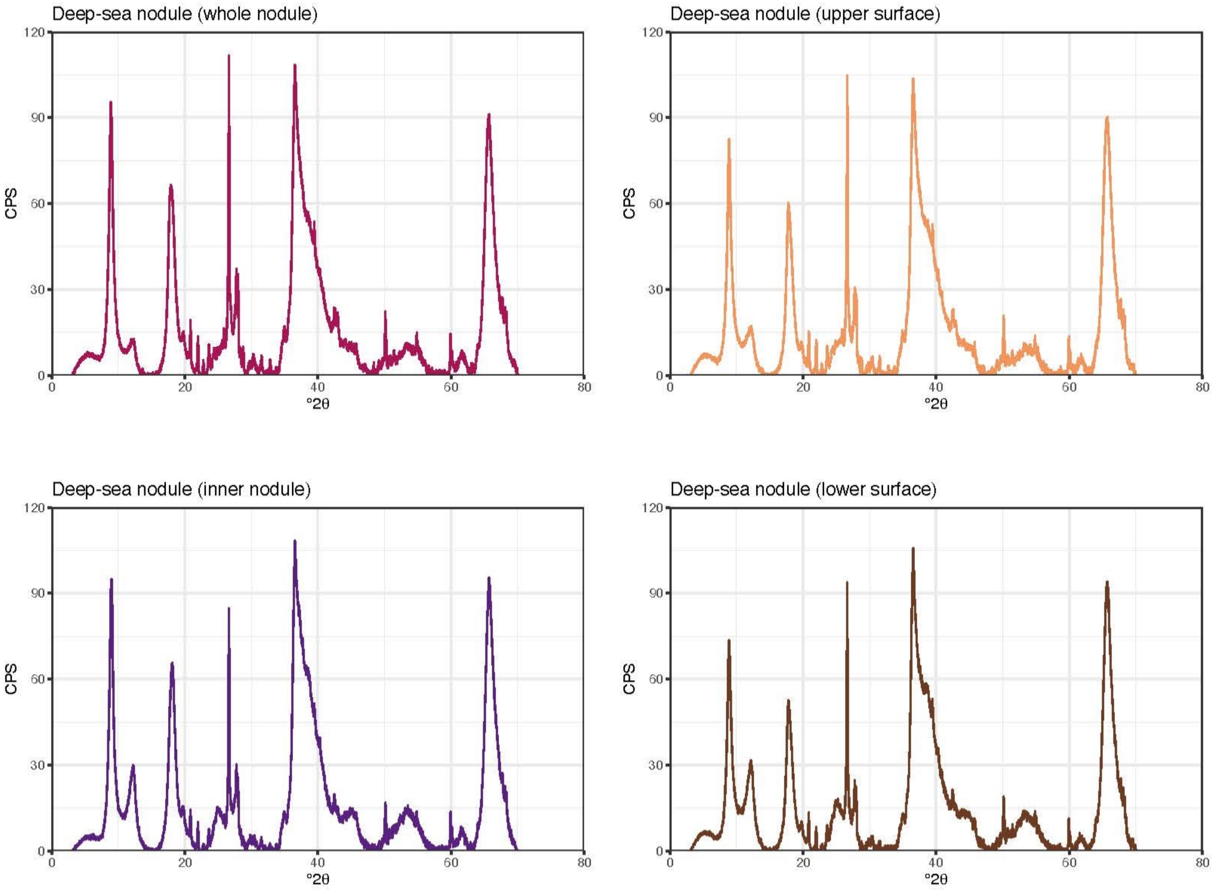

Appendix 2: Information on push-core samples taken after decompaction in an old epibenthic sledge track with a “decompaction rake” in the BGR/GER polymetallic nodule exploration area in the CCZ in 4127 meters water depth. Station number, date of sampling, ROV dive number (#), latitude, longitude, PUC core number, fixative, research purpose and comments are provided. F = Formaldehyde; env vars = environmental variables.

Station number	Date Sampling	ROV Dive #	Latitude	Longitude	Core#	Fixative	Research Purpose	Comment
SO268/2_188_1	12/5/2019	29	11° 51.617' N	117° 00.747' W	PUC 20	frozen	env vars	decompaction stretch 1
SO268/2_188_1	12/5/2019	29	11° 51.617' N	117° 00.747' W	PUC 7	4% F	community	decompaction stretch 1
SO268/2_188_1	12/5/2019	29	11° 51.617' N	117° 00.747' W	PUC 27	4% F	community	control 1
SO268/2_188_1	12/5/2019	29	11° 51.617' N	117° 00.747' W	PUC 71	frozen	env vars	control 1
SO268/2_188_1	12/5/2019	29	11° 51.617' N	117° 00.747' W	PUC 24	4% F	community	decompaction stretch 2
SO268/2_188_1	12/5/2019	29	11° 51.617' N	117° 00.747' W	PUC 36	frozen	env vars	decompaction stretch 2
SO268/2_188_1	12/5/2019	29	11° 51.617' N	117° 00.747' W	PUC 11	4% F	community	control 2
SO268/2_188_1	12/5/2019	29	11° 51.617' N	117° 00.747' W	PUC 57	frozen	env vars	control 2
SO268/2_188_1	12/5/2019	29	11° 51.617' N	117° 00.747' W	PUC 46	frozen	env vars	decompaction stretch 3
SO268/2_188_1	12/5/2019	29	11° 51.617' N	117° 00.747' W	PUC 12	4% F	community	decompaction stretch 3
SO268/2_188_1	12/5/2019	29	11° 51.617' N	117° 00.747' W	PUC 17	4% F	community	control 3
SO268/2_188_1	12/5/2019	29	11° 51.617' N	117° 00.747' W	PUC 25	frozen	env vars	control 3

Appendix 3: Overview of bar plots (average ± standard deviation) for all environmental variables (granulometry with median grain size, %sand, %silt, %clay; nutrients with total organic carbon (TOC), % total nitrogen (TN), C:N) between decompacted samples and control samples per sediment depth (0-1 cm, 1-2cm, 2-3cm, 3-4cm, 4-5cm).

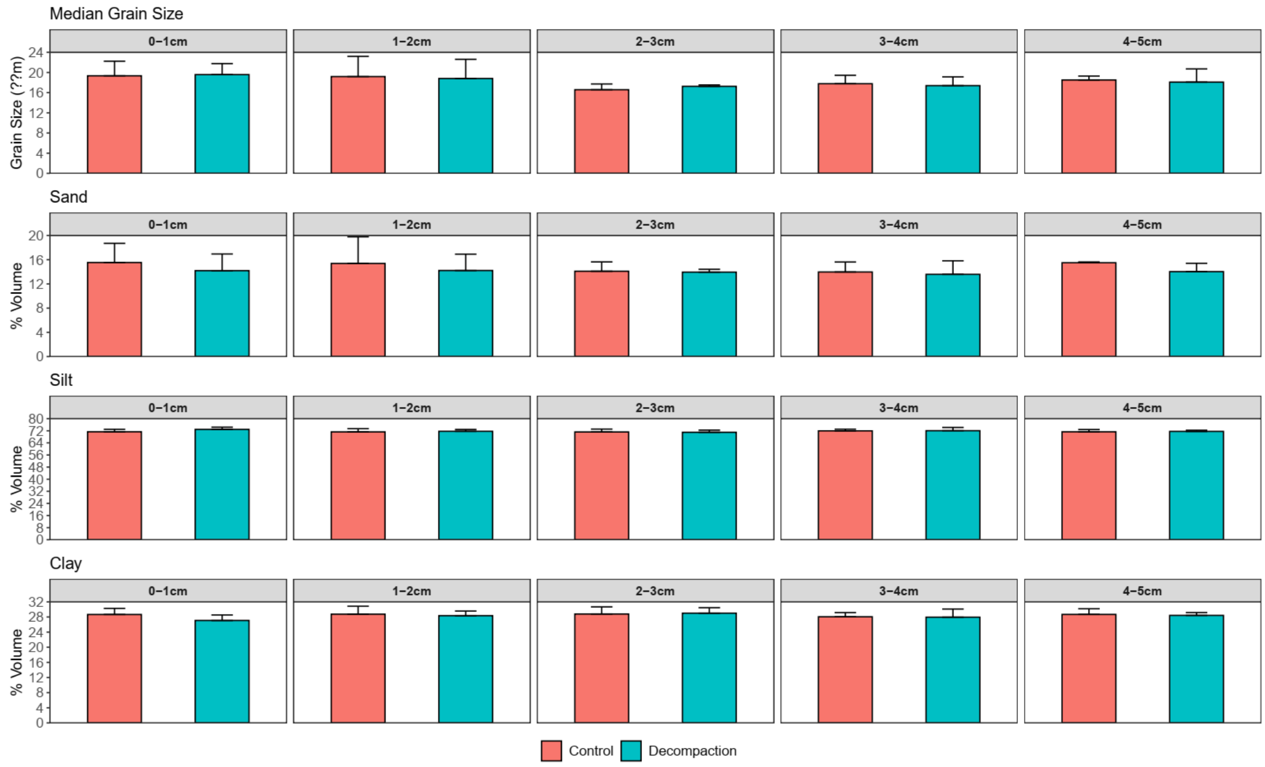

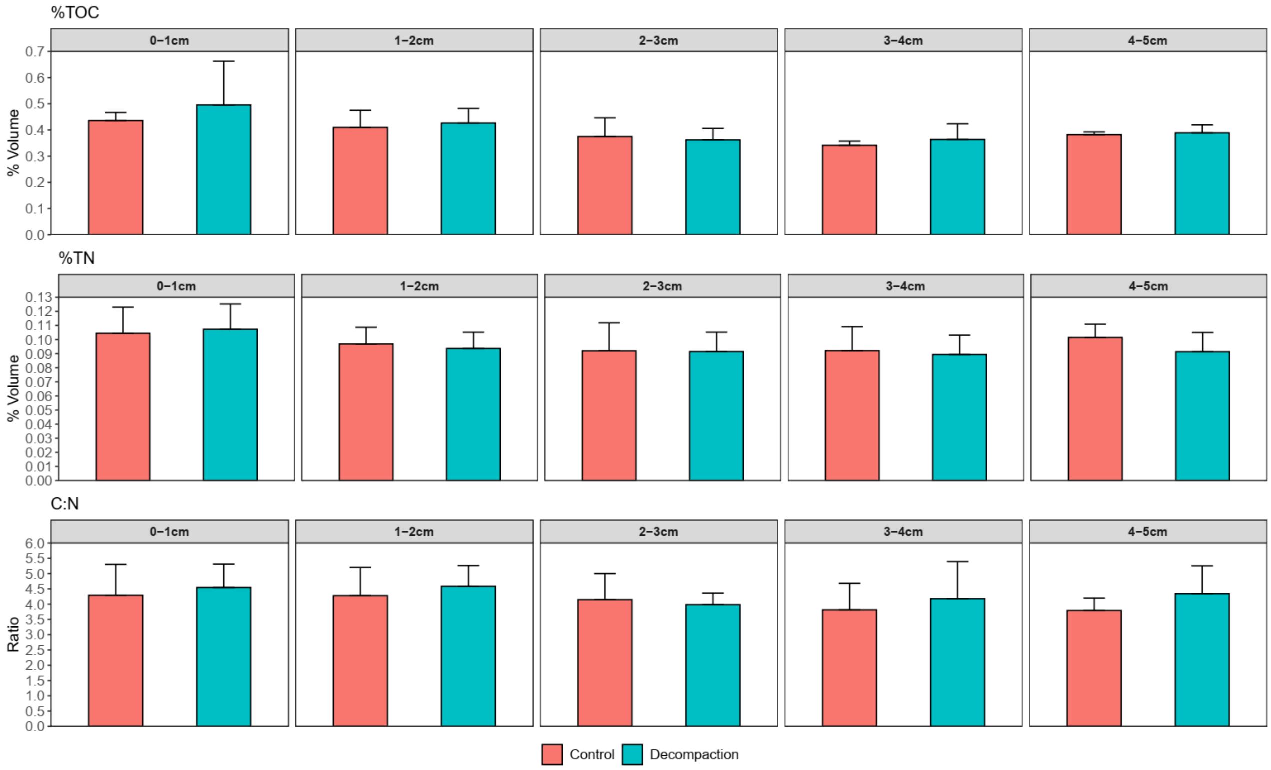

Supplement: Supplementary file 1 — Three appendices with information on (1) figures on the mineral content of artificial and natural nodules, (2) table on push‐core samples collected in the decompaction area, and (3) figures on environmental variables measured in the decompaction area. [file IEAM-18-682-s001.pdf]
